# Optimization and HPLC-DAD Characterization of Phenolic Compounds and Antioxidant Activity in *Ferula communis* L. Inflorescence

**DOI:** 10.3390/molecules31132235

**Published:** 2026-06-25

**Authors:** Mounya Laachir, Nora Ouyahya, Mouhcine Fadil, Khaoula Faiz, Mohammed Merzouki, Lahsen El Ghadraoui, Karima Mikou

**Affiliations:** 1 Functional Ecology and Environmental Engineering Laboratory, Faculty of Sciences and Techniques, Sidi Mohamed Ben Abdellah University, Fez P.O. Box 2202, Morocco; nora.ouyahya@usmba.ac.ma (N.O.);; 2 Laboratory of Applied Organic Chemistry, Faculty of Sciences and Techniques, Sidi Mohamed Ben Abdellah University, Fez P.O. Box 2202, Morocco; 3 Biotechnology, Environment, Agri-Food and Health Laboratory, Faculty of Sciences Dhar El Mahraz, Sidi Mohamed Ben Abdellah University, Fez P.O. Box 2202, Morocco

**Keywords:** optimization, *Ferula communis* inflorescence, phenolic compounds, response surface methodology, antioxidant activity

## Abstract

An ultrasound-assisted extraction method combined with response surface methodology was applied to optimize the extraction of phenolic compounds and evaluate the antioxidant capacity of *Ferula communis* inflorescence. Response surface methodology with a central composite design was used to optimize the process. As independent variables, ethanol concentration (40–80% *v*/*v*), solvent-to-sample ratio (10–40 mL/g), and extraction time (14–30 min) were assessed. A solid-to-liquid ratio of 19 mL/g, a 60% ethanol concentration, and a 23 min extraction duration were the ideal extraction parameters. The extraction yield, total phenolic content (TPC), total flavonoid content (TFC), and DPPH radical scavenging capacity (IC_50_) were predicted to be 18.91%, 34.93 mg GAE/g, 18.03 mg QE/g, and 0.58 mg/mL, respectively, under these optimized conditions. The efficacy of the central composite design in optimizing polyphenol extraction from *F. communis* was validated by experimental results that closely matched the predicted values. Eleven phenolic compounds were identified by HPLC-DAD, with pyrogallol and kaempferol being the most prevalent components. These results suggest that *F. communis* may represent a promising source of bioactive compounds with potential antioxidant applications.

## 1. Introduction

The genus *Ferula*, belonging to the Apiaceae family, comprises approximately 180–185 species and is widely distributed from the Mediterranean region to Central Asia [1,2]. In Morocco, six *Ferula* species have been observed—*F. communis*, *F. cossoniana*, *F. gouliminensis*, *F. sauvagei*, *F. atlantica*, and *F. tingitana*—with different and variable geographic distributions [3]. The accumulation of oleo-gum-resins in the roots and stems of *Ferula* species gives them a strong bitter taste and strong odor, which is mostly caused by sulfur-containing substances and other bioactive phytochemicals [4].

*Ferula communis* L. (giant fennel), locally known as “Elboubale”, “Fassough” or “Ouffale”, is an abundant latex-producing perennial reaching 1 to 3 m in height. The species occurs in two chemotypes: a toxic form rich in ferulenol, responsible for anticoagulant and hemorrhagic effects; and a non-toxic form containing ferutinin, a sesquiterpene ester with estrogenic activity. *F. communis* is of considerable biological and therapeutic interest. It has been demonstrated that this plant exhibits biological activities such as antioxidant, antibacterial, antifungal, and anticancer effects [5,6,7]. These activities vary depending on the chemical composition of the extracts from the part of the plant used [8,9]. Furthermore, it has been demonstrated that *F. communis* is characterized by its presence of flavonoids and polyphenols [10]. These bioactive molecules have natural antioxidant potential [11,12]. Although *F. communis* is a promising source of bioactive compounds, this species remains insufficiently scientifically explored, particularly with regard to optimizing extraction conditions, detailed characterization of its phenolic compounds, and evaluation of its biological activities. Most phytochemical and biological studies conducted on *F. communis* have primarily focused on the roots, which are rich in secondary metabolites. In contrast, other plant organs, such as stems, seeds, and particularly inflorescences, remain poorly characterized from a biochemical perspective, representing a gap in the current literature on this species. Furthermore, conventional extraction methods, such as maceration and Soxhlet extraction present several limitations in terms of extraction efficiency, solvent consumption, and the preservation of thermolabile bioactive compounds. Ultrasound-assisted extraction (UAE) represents a particularly effective alternative, as it enhances the extraction yield of phenolic compounds while reducing extraction time and energy consumption. However, this method requires a rigorous statistical approach. Hence the use of Response Surface Methodology (RSM), which enables the simultaneous optimization of multiple independent variables and the modeling of their interactions.

To the best of our knowledge, this study represents one of the few attempts to optimize the extraction of phenolic compounds from the inflorescence of *F. communis* using a combined UAE–RSM approach. The novelty of this work lies in (i) the valorization of a relatively underexplored plant organ from a species of high medicinal interest, (ii) the application of a rigorous optimization methodology, and (iii) the development of a predictive model for the production of phenolic-rich extracts from this species.

In this context, the present study aims to optimize extraction conditions in order to maximize the yield of bioactive compounds, as well as to evaluate the antioxidant activity of the extracts, with a view to their potential use in the cosmetic, pharmaceutical, and medicinal fields. To this end, a Central Composite Design (CCD) experimental plan, coupled with Response Surface Methodology (RSM), was applied to study the combined effect of three key variables, namely extraction time, solvent-to-solid ratio (mL/g), and ethanol concentration (%), on the yield of phenolic compounds and antioxidant activity. In addition, the phenolic compounds were quantified and characterized using high-performance liquid chromatography coupled with a diode array detector (HPLC-DAD), enabling qualitative and quantitative analysis of the phenolic profile of the optimized extracts.

## 2. Results

### 2.1. Experimental Design Matrix and Results

The experimental plan and the corresponding observed values for each response are presented in Table 1.

### 2.2. Analysis of Variance (ANOVA) and Model Fitting

The statistical significance and adequacy of the developed polynomial models were evaluated using analysis of variance (ANOVA). The results are summarized in Table 2.

**Figure 1:**
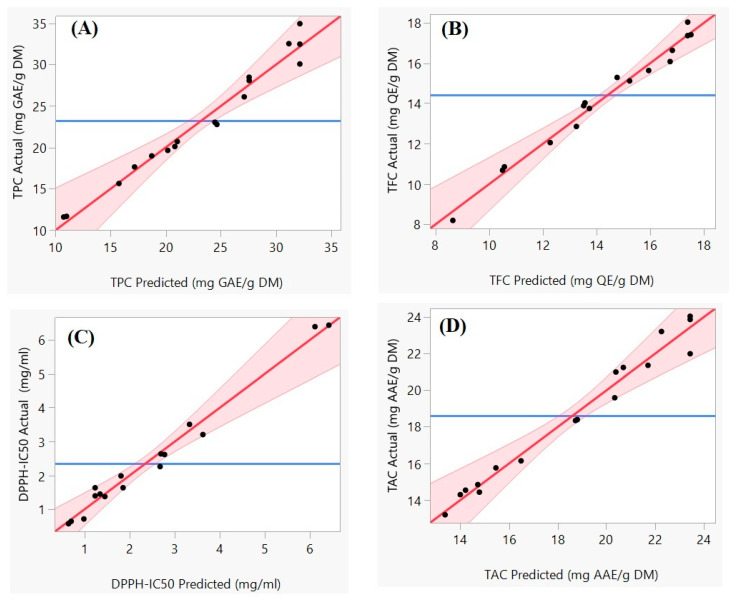
Plots of actual vs. predicted values for (**A**) TPC, (**B**) TFC, (**C**) DPPH-IC50, and (**D**) TAC.

The ANOVA results indicate that the regression models for all four responses are highly significant, as evidenced by their low *p*-values (<0.0001). Furthermore, the lack of fit test was used to assess the model’s adequacy. The *p*-values for lack of fit (0.81, 0.13, 0.09, and 0.77) are all greater than 0.05, indicating that the lack of fit is not significant relative to the pure error. This confirms that the developed second-order models are adequate and robust for predicting the responses within the experimental domain. The high coefficients of determination (R^2^) (0.97, 0.98, 0.98, and 0.97 for TPC, TFC, DPPH-IC50, and TAC, respectively) confirm an excellent correlation between the experimental data and the predicted values. To further validate the robustness of the models, the adjusted R^2^ (R^2^_adj_) was evaluated. The models yielded high R^2^_adj_ values of 0.93 (TPC), 0.96 (TFC), 0.95 (DPPH-IC50), and 0.94 (TAC), which are very close to their corresponding R^2^ values, indicating that the models are highly significant. Additionally, the predictive capability of the models for new observations was assessed using the predicted R^2^ (R^2^_pred_), which were found to be 0.85, 0.77, 0.85, and 0.86 for TPC, TFC, DPPH-IC50, and TAC, respectively. For all four responses, the reasonable agreement between R^2^_adj_ and R^2^_pred_ (a difference of less than 0.20) mathematically substantiates the high predictive power of the developed second-order models. The goodness-of-fit for each model was visually confirmed by plotting the actual (experimental) values against the model-predicted values (Figure 1). The repeatability of the ultrasound-assisted extraction process and the magnitude of pure experimental error were evaluated using the central point replicates (Runs 15, 16, and 17 in Table 1). While slight variations are inherently expected when extracting bioactive compounds from complex and heterogeneous biological matrices such as plant inflorescences, the variation remained strictly within acceptable limits. For instance, the Relative Standard Deviation (RSD) for the Total Phenolic Content (TPC) at the central point was calculated to be 7.48%, with TFC, DPPH-IC50, and TAC showing RSDs of 2.2%, 9.0%, and 4.9%, respectively. Because these values are below the generally accepted threshold of 10% for phytochemical extractions, the method demonstrates good repeatability.

As shown in Figure 1, the data points are tightly clustered around the 45° line, indicating a strong linear relationship between observed and predicted values. This provides further confirmation of the high predictive power of the models, in agreement with the high R^2^ values.

### 2.3. Analysis of Factor Effects and Regression Models

The estimated regression coefficients, along with their statistical significance (*p*-values), are detailed in Table 3. These coefficients quantify the effect of each factor and their interactions on the responses. The final predictive equations were constructed by retaining only the statistically significant terms (*p* < 0.05) from the full second-order polynomial model. No formal model reduction was performed, as the full model already demonstrated excellent adequacy (non-significant lack of fit, R^2^ > 0.97 for all responses). Table 3 therefore reports the complete set of estimated coefficients from the full model, with non-significant terms explicitly identified, allowing for the direct identification of the factors and interactions that significantly contribute to each response.

### 2.4. Model for Total Phenolic Content (TPC)

The significant terms (*p* < 0.05) are Intercept (β_0_), Time (β_1_), ETOH Conc. (β_2_), and the quadratic terms Time^2^ (β_11_), ETOH Conc.^2^ (β_22_), and S/M Ratio^2^ (β_33_). The final predictive equation in terms of coded factors is:

Y_TPC_ = 32.15 + 1.55X_1_ + 3.26X_2_ − 6.15X_1_^2^ − 4.26X_2_^2^ − 4.82X_3_^2^ (1)

The linear effects showed that extraction time (β_1_ = +1.55) and ethanol concentration (β_2_ = +3.26) exerted significant positive influences on total phenolic content, with the ethanol concentration being the most influential factor, indicating that increasing these parameters initially enhances phenolic extraction, whereas the solvent-to-material ratio (β_3_) did not present a significant linear effect (*p* > 0.05). In contrast, none of the interaction terms (β_12_, β_13_, β_23_) were statistically significant, suggesting that the extraction factors act independently on TPC, which simplifies the optimization process. Furthermore, all quadratic terms (β_11_, β_22_, β_33_) were significant and strongly negative, revealing a pronounced curvature in the response surface and confirming the existence of optimal levels for each factor, beyond which further increases in extraction time, ethanol concentration, or solvent-to-material ratio lead to a reduction in TPC yield.

### 2.5. Model for Total Flavonoid Content (TFC)

The significant terms are Intercept (β_0_), Time (β_1_), ETOH Conc. (β_2_), the interaction Time ∗ S/M Ratio (β_13_), and the quadratic terms Time^2^ (β_11_) and ETOH Conc.^2^ (β_22_). The equation is:

Y_TFC_ = 17.39 + 1.61X_1_ + 1.10X_2_ − 0.79X_1_X_3_ − 2.26X_1_^2^ − 2.55X_2_^2^ (2)

The linear effects indicated that, similarly to TPC, extraction time (β_1_ = +1.61) and ethanol concentration (β_2_ = +1.10) had significant positive influences on total flavonoid content, thereby enhancing flavonoid extraction. A significant and negative interaction was observed between extraction time and the solvent-to-material ratio (β_13_ = −0.79), revealing an antagonistic effect whereby the positive impact of increasing extraction time is reduced at higher solvent-to-material ratios, and vice versa. In addition, the quadratic terms for time^2^ (β_11_ = −2.26) and ethanol concentration^2^ (β_22_ = −2.55) were both significant and negative, indicating the presence of optimal levels for these factors, beyond which further increases lead to a decrease in flavonoid extraction efficiency.

### 2.6. Model for DPPH-IC50

The significant terms are Intercept (β_0_), Time (β_1_), ETOH Conc. (β_2_), S/M Ratio (β_3_), the interaction Time ∗ ETOH Conc. (β_12_), and the quadratic terms Time^2^ (β_11_) and ETOH Conc.^2^ (β_22_). The equation is:

Y_DPPH-IC50_ = 1.24 − 0.61X_1_ − 1.49X_2_ + 0.43X_3_ + 1.01X_1_X_2_ + 0.83X_1_^2^ + 0.90X_2_^2^ (3)

The linear effects showed that extraction time (β_1_ = −0.61) and ethanol concentration (β_2_ = −1.49) had significant negative effects, which is desirable, as increasing these factors leads to lower IC_50_ values, indicating higher antioxidant capacity, whereas the solvent-to-material ratio (β_3_ = +0.43) had a significant positive effect, suggesting that higher solvent ratios may lead to a decrease in antioxidant performance. A significant and positive interaction was observed between time and ethanol concentration (β_12_ = +1.01), revealing a strong antagonistic effect in which the combined increase in both factors partially offsets their individual benefits, highlighting the need for an optimal balance. Additionally, the quadratic terms for time^2^ (β_11_ = +0.83) and ethanol concentration^2^ (β_22_ = +0.90) were significant and positive, indicating that exceeding the optimal levels of these factors result in an increase in IC_50_, thereby diminishing antioxidant activity.

### 2.7. Model for Total Antioxidant Capacity (TAC)

The significant terms are Intercept (β_0_), Time (β_1_), ETOH Conc. (β_2_), and all three quadratic terms (β_11_, β_22_, β_33_). The equation is:

Y_TAC_ = 23.43 + 0.79X_1_ − 0.96X_2_ − 3.84X_1_^2^ − 2.14X_2_^2^ − 2.24X_3_^2^ (4)

The linear effects showed that extraction time (β_1_ = +0.79) had a significant positive influence on total antioxidant capacity (TAC), indicating that longer extraction times favor higher TAC values, whereas ethanol concentration (β_2_ = −0.96) exerted a significant negative effect. This contrasting behavior suggests that increasing ethanol concentration reduces TAC, implying that the compounds mainly responsible for total antioxidant capacity are preferentially extracted using more polar solvents with lower ethanol content, in direct contrast to the trends observed for TPC and TFC. No interaction terms were statistically significant, indicating independent effects of the studied factors on TAC. Furthermore, all quadratic terms (β_11_, β_22_, β_33_) were significant and negative, confirming the existence of optimal levels for each factor beyond which TAC decreases.

### 2.8. Parameter Optimization

#### 2.8.1. Response Surface and Contour Plot Analysis

To visualize the effects of the factors and identify optimal regions, 2D contour plots and 3D response surfaces were generated. For clarity, the response was plotted against the two most influential factors (Time and ETOH Concentration), while the third factor (S/M Ratio) was held constant at its optimal level for each specific response.

**Figure 2:**
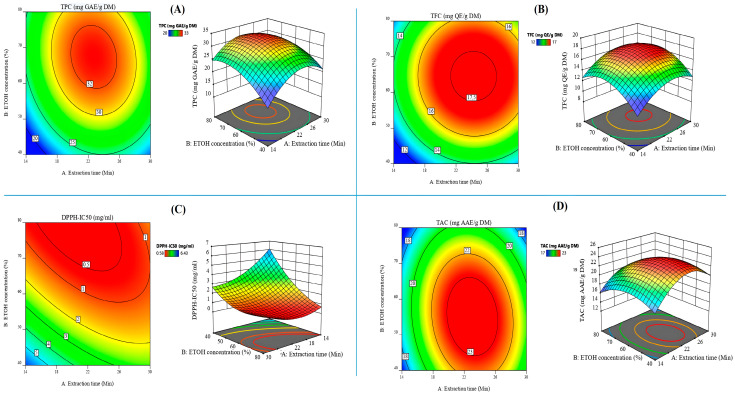
2D contour plots and 3D response surface plots for the four responses—TPC (**A**), TFC (**B**), DPPH-IC50 (**C**), and TAC (**D**)—as a function of extraction time and ethanol concentration, with the solvent-to-material ratio held constant at its optimal value for each response.

Based on the analysis of these plots (Figure 2), the following optimal ranges were identified from the response surface models for each individual response, For total phenolic content (TPC), an optimal yield of approximately 32 mg GAE/g DW was achieved using a solvent-to-material ratio of 21, an extraction time ranging between 19 and 25 min, and an ethanol concentration between 58% and 76%. Concerning total flavonoid content (TFC), a maximum yield of about 17.5 mg QE/g DW was obtained at a solvent-to-material ratio of 24, an extraction time between 21 and 27 min, and an ethanol concentration ranging from 57% to 71%. Regarding antioxidant activity evaluated by the DPPH assay, a minimum IC_50_ value of approximately 0.5 mg/mL was achieved using a solvent-to-material ratio of 10, an extraction time between 15 and 26 min, and an ethanol concentration between 66% and 80%. Finally, the total antioxidant capacity (TAC) reached approximately 23 mg AAE/g DW under optimal conditions corresponding to a solvent-to-material ratio of 19, an extraction time ranging from 18 to 26 min, and an ethanol concentration between 44% and 66%. These intervals represent model-based predictions derived from the fitted second-order polynomials.

#### 2.8.2. Desirability Function Analysis for Individual Optimization

**Figure 3:**
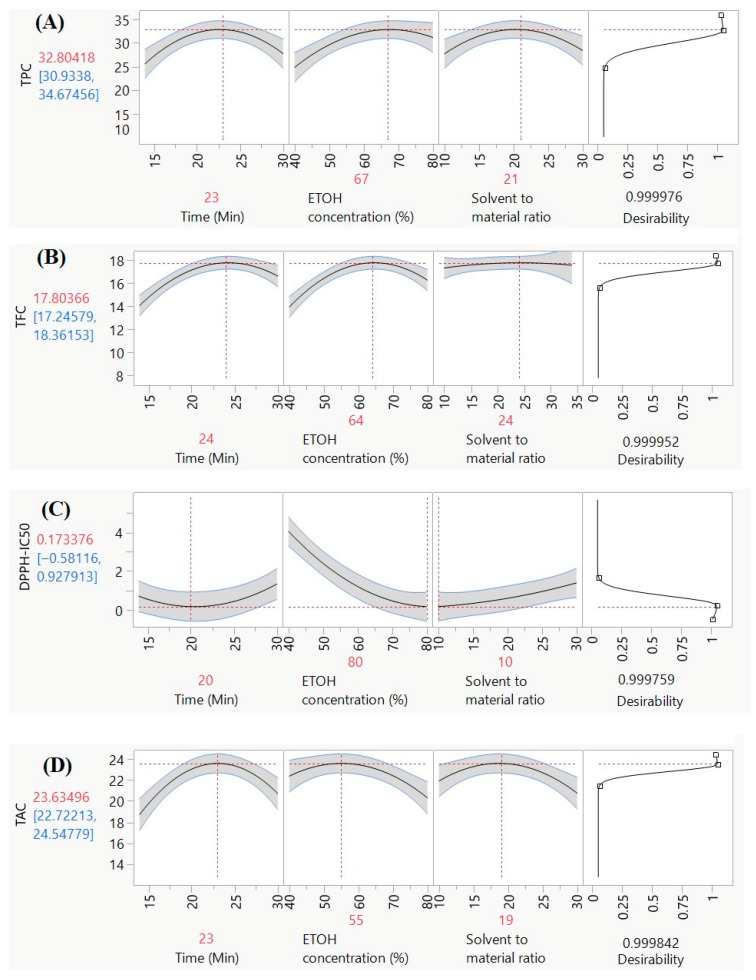
Desirability profiles for the individual optimization of the four responses: TPC (**A**), TFC (**B**), DPPH-IC50 (**C**), and TAC (**D**).

To precisely determine the optimal conditions, a desirability function analysis was performed. Figure 3 shows the desirability profiles for optimizing each response individually.

The optimal extraction conditions leading to a desirability score close to 0.99 were determined for each response. The maximum total phenolic content (TPC) of 32.8 mg GAE/g DW was achieved at an extraction time of 23 min, an ethanol concentration of 67%, and a solvent-to-material ratio of 21. Similarly, the total flavonoid content (TFC) was maximized to 17.8 mg QE/g DW under conditions of 24 min extraction time, 64% ethanol, and a solvent-to-material ratio of 24. Regarding antioxidant activity, the lowest DPPH IC_50_ value (0.17 mg/mL) was obtained at an extraction time of 20 min, with 80% ethanol and a solvent-to-material ratio of 10. Finally, the total antioxidant capacity (TAC) reached a maximum value of 23.6 mg AAE/g DW when the extraction was performed for 23 min using 55% ethanol and a solvent-to-material ratio of 19.

#### 2.8.3. Simultaneous (Multi-Response) Optimization

The individual optimization results show a conflict in the optimal conditions. Specifically, minimizing DPPH-IC50 requires a high ethanol concentration (80%) and a low solvent ratio (10), whereas the other three responses (TPC, TFC, TAC) are optimized under moderate conditions (55–67% ethanol, ratio of 19–24).

**Figure 4:**
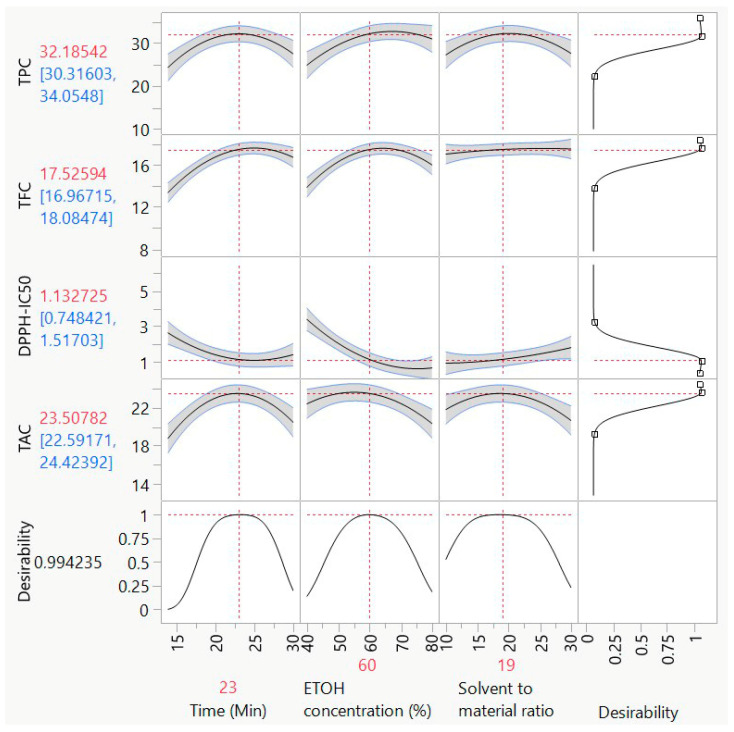
Desirability profile for the simultaneous optimization of the four responses (TPC, TFC, DDPH-IC50, and TAC).

Therefore, a simultaneous optimization was conducted using the desirability tool to find a single set of compromise conditions that would provide the best possible outcome across all four responses. The desirability profile for this multi-response optimization is shown in Figure 4. While this approach is highly beneficial for TPC, TFC, and TAC, it results in a slight compromise (a less-than-ideal value) for the DPPH-IC50 response.

The optimal conditions for the simultaneous optimization of all four responses were determined as an extraction time of 23 min, an ethanol concentration of 60%, and a solvent-to-material ratio of 19. Under these conditions, a composite desirability of 0.994 was achieved, indicating the best compromise for maximizing phenolic and flavonoid contents as well as total antioxidant capacity, while maintaining strong radical scavenging activity.

#### 2.8.4. Experimental Validation of the Optimal Conditions

To evaluate the predictive accuracy of the established models, an experimental validation was conducted for the four targeted responses (TPC, TFC, DPPH-IC50, and TAC). These confirmation tests were performed using the simultaneous optimal conditions derived from the RSM analysis: an extraction time of 23 min, 60% ethanol, and a solvent-to-material ratio of 19. As shown in Table 4, the experimental data closely matched the model-predicted values. This strong correlation between the theoretical and observed results verifies the reliability and robustness of the mathematical approach. Ultimately, these findings confirm that the RSM models are both statistically valid and highly practical for predicting how extraction parameters influence the recovery of bioactive compounds.

### 2.9. Phytochemical Analysis of F. communis Extract

**Figure 5:**
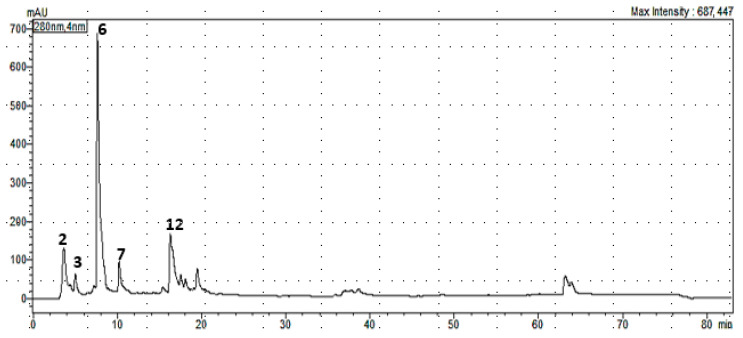
HPLC-DAD chromatogram of *F. communis* ethanolic extract and standards at 280 nm, showing the main identified phenolic compounds: (**2**) pyrogallol, (**3**) gallic acid, (**6**) caffeic acid, (**7**) kaempferol, and (**12**) ferulic acid.

Using HPLC-DAD, eleven phenolic compounds were identified and quantified in the inflorescence of *F. communis*, namely: pyrogallol, gallic acid, catechol, kaempferol, protocatechuic acid, syringic acid, coumaric acid, ferulic acid, vanillic acid, rutin, and quercetin (Figure 5).

The identification of compounds was performed by comparing their retention times with those of authentic standards. As shown in Table 5, the extract was characterized by the presence of pyrogallol, gallic acid, catechol, protocatechuic acid, syringic acid, coumaric acid, ferulic acid, and vanillic acid, along with flavonoids such as kaempferol, rutin, and quercetin. Among the detected compounds, kaempferol exhibited the highest concentration, followed by pyrogallol, rutin, and ferulic acid.

## 3. Discussion

### 3.1. Phytochemical Composition

The optimization of extraction conditions suggests that intermediate extraction time, moderate ethanol concentration, and an appropriate solvent-to-solid ratio favor the efficient recovery of polyphenols from the plant material. These three parameters have a significant impact on extraction yield; however, some degree of interaction between them may exist, highlighting the importance of optimizing them in combination to maximize phenolic compound content while reducing extraction time and solvent consumption. Ref. [13] have confirmed the benefits of combining several factors, including the use of ethanol solutions, time optimization, and the ultrasonic extraction process at *Centella asiatica*. This method has made it possible to obtain particularly high levels of phenolic compounds and flavonoids. Furthermore, although several studies have addressed the optimization of polyphenol extraction, limited information is available regarding its application in *Ferula* species, which may justify further investigation.

Our results show that the maximum yield of phenolic compounds, using optimal extraction conditions (67% ethanol, 23 min extraction time, and a solid–solvent ratio of 1:21), is in the range of 32.8 mg GAE/g DW and 17.8 mg QE/g DW for polyphenols and flavonoids, respectively. Similar levels have been reported in plants such as *Rhus typhina* [14] and *Phyllanthus emblica* [15], which are recognized for their richness in phenolic compounds and their significant antioxidant and antimicrobial activities.

Analysis of our results reveals that the extraction of polyphenols and flavonoids is influenced by the nature of the solvent used and by the percentage of ethanol in the hydroalcoholic extraction solvent; this percentage determines the solubility of polyphenols through its effect on the polarity of the extraction medium. Thus, the highest yields were obtained when the hydroalcoholic mixture had intermediate polarities between 60 and 67%, combined with a solid–liquid ratio of 1/20. At this concentration, the solvent promotes the solubilization of phenolic compounds with different polarities. Polyphenols, which have a wide variety of structures and polarities, are best extracted with a hydroalcoholic solvent that allows both slightly hydrophilic and slightly hydrophobic molecules, such as flavonoids, to be extracted efficiently.

*F. communis* leaves polyphenol content (91.5 ± 3.01 mg GAE/g) was observed in [10] and is significantly higher than that obtained for the inflorescence in the present study (34 mg GAE/g). This is consistent with data in the literature reporting heterogeneity in the distribution of metabolites within different plant organs [16]. Leaves are generally richer in secondary metabolites than other organs. This is because leaves, which are directly exposed to UV rays, oxidation, and environmental stress, tend to concentrate higher levels of phenolic compounds to defend themselves against adverse conditions. Furthermore, the high content of secondary metabolites in leaves in general, and phenolic compounds in particular, can be explained by their high photosynthetic activity, which provides the precursors necessary for the synthesis of secondary metabolites. Finally, the relatively low polyphenol content of inflorescence could be linked to their function, which is more oriented towards the synthesis of pigments and attractive compounds in order to better ensure pollination.

### 3.2. Antioxidant Activity of the Inflorescence of F. communis

We demonstrated the antioxidant activity of the extracts using two different tests: the DPPH test and the TAC test. The anti-radical capacity measured by DPPH (IC_50_ = 0.17 mg/mL) was obtained with a hydroalcoholic solvent composed of 80% ethanol. With the TAC test, the antioxidant capacity measured is 23.6 mg AAE/g DW obtained with a 55% ethanolic extract. This difference can be explained by the nature of the tests used. The DPPH assay evaluates the ability of a compound to scavenge free radicals through electron and hydrogen atom donation, leading to their neutralization. This activity is mainly related to molecular structure, particularly the presence of phenolic groups, and is often observed in compounds soluble in low-polarity solvents. In contrast, the TAC test, which measures the total antioxidant capacity of the extract, involves more polar compounds that are better extracted at lower alcohol concentrations, such as in a 55% ethanol solvent.

Furthermore, our results also showed that the solvent that yielded the highest polyphenol content (67% ethanol) was not the one with the best antioxidant activity. This can be explained by the fact that the antioxidant activity of polyphenols varies depending on their chemical structure and their ability to interact with free radicals or oxidizing agents. In addition, the antioxidant activity observed may be attributed to other non-phenolic antioxidant compounds. Others [17] have shown in other species that the polarity of the solvent used plays a crucial role in the recovery of bioactive compounds and in the biological activities of the extracts.

The strong antioxidant activity observed in this study can be mainly attributed to the presence of powerful radical scavengers such as gallic acid and pyrogallol. In addition, flavonoids such as kaempferol may contribute significantly to the overall antioxidant capacity through electron transfer mechanisms, suggesting a synergistic effect among different classes of phenolic compounds.

Finally, all these results highlight the importance of an optimization approach that includes both the yield of bioactive compounds and the biological activity of the extracts.

### 3.3. Phytochemical Analysis of the Extract

Qualitative and quantitative analysis of the hydroethanolic extract of *F. communis* inflorescence, performed by HPLC-DAD, enabled us to identify 11 phenolic compounds, including tannic acid, pyrogallol, gallic acid, catechol, hydroxytyrosol, caffeic acid, as well as numerous aromatic acids, such as protocatechuic acid, syringic acid, p-coumaric acid, ferulic acid, and vanillic acid. To the best of our knowledge, this is the first study reporting the identification of these phenolic compounds in the inflorescence of *F. communis.*

These compounds identified in our extract belong to two different families: those of hydroxybenzoic acids represented by gallic, protocatechuic, syringic, vanillic, tannic, pyrogallol, and catechol acids; and hydroxycinnamic acids represented by caffeic, p-coumaric, and ferulic acids. The simultaneous presence of these two families shows that the studied extract has a very significant chemical richness, which may be associated with relevant bioactive properties of the extract, supporting its potential applications in different sectors such as cosmetics and agri-food. Indeed, beyond the well-documented antioxidant properties of these compounds [10]. Other potential properties have been attributed to it, such as antimicrobial, anti-inflammatory, astringent, anti-cancer, major antioxidant, and neuroprotective properties [5,6,11].

Our analyses show that *Ferula* inflorescence is an attractive source of bioactive compounds (phenols and flavonoids), which are frequently associated with antioxidant, antimicrobial, and even enzyme-inhibiting properties, hence the interest in pharmaceutical, nutraceutical, or cosmetic applications of extracts from the plant studied, especially since other important metabolites have been identified in other organs of this plant. Ref. [5] reported the presence of bioactive sesquiterpenes with notable antibacterial properties, such as ferulene, ferchromone, elemicin, feselol, and daucane ester, in the rhizomes of *F. communis*, confirming the potential of this species.

## 4. Material and Methods

### 4.1. Sample Preparation

Inflorescence of *F. communis* was collected in March 2025 from Taounate city in Morrocco. Mr. Eloutassi Noureddine, from the Laboratory of Engineering, Electrochemistry, Modeling and Environment, Faculty of Sciences Dhar El Mahraz, Sidi Mohamed Ben Abdellah University (USMBA), Fez, Morocco, carried out the botanical identification. A voucher specimen was deposited under the reference number FAA/120325/01. The aerial parts were washed, shade-dried at room temperature to constant weight for seven days, ground using an electric grinder and sieved to obtain a fine powder. The resulting powders were stored at 4 °C until further analysis. The extraction was carried out using ultrasound-assisted extraction (UAE), by varying three experimental parameters: ethanol concentration, solid-to-solvent ratio (*w*/*v*), and sonication time. The extraction was carried out using a VWR USC 300 TH ultrasonic cleaner bath operating at a power of 200 W and a frequency of 45 kHz. During this ultrasound-assisted extraction, three experimental parameters were varied: ethanol concentration, solid-to-solvent ratio (*w*/*v*), and sonication time. This methodological choice is supported by the well-established efficiency of ultrasound-assisted extraction in releasing phenolic compounds and other bioactive metabolites [18].

### 4.2. Determination of Total Phenolic Content (TPC)

The Folin–Ciocalteu reagent was used to determine the total phenolic content concentration as described by [19] with modifications. Briefly, 200 μL of each extract was mixed with 1000 μL of 10% Folin–Ciocalteu reagent and incubated for 5 min at room temperature (25 ± 2 °C). After neutralization with 800 μL of 7.5% sodium carbonate solution, the mixture was incubated for an additional 2 h. The absorbance was measured at 765 nm using a UV–visible spectrophotometer. Gallic acid (0–100 μg/mL) was used as a standard to produce a linear calibration curve. The obtained results were expressed as mg gallic acid equivalents per gram (mg GAE/g DW).

### 4.3. Determination of Total Flavonoid Content (TFC)

The total flavonoid content of the extracts was determined by a colorimetric method using aluminum chloride [20]. Briefly, 0.25 mL of the extract was mixed with 75 µL of 5% NaNO_2_ and 150 µL of a 10% methanolic AlCl_3_ solution. After 6 min, NaOH (1 M) was added to the mixture and the final volume was adjusted to 3 mL with distilled water. The absorbance was then determined at 510 nm. A quercetin standard calibration curve was used to carry out the quantification, and the findings were represented as mg of quercetin equivalents per gram of extract (mg QE/g).

### 4.4. Antioxidant Activity

#### 4.4.1. DPPH Radical Activity Scavenging Assay

The antioxidant activity of *F. communis* extract was tested for its ability to neutralize the 2,2-diphenyl-1-picrylhydrazyl (DPPH) radical [21]. Briefly, 1975 μL of a 60 µM methanolic DPPH solution were mixed with 25 µL of each extract. Following a half-hour incubation period in the dark at a temperature of 14.0 ± 2.0 °C, the mixture’s absorbance at 517 nm was determined. The following formula was used to calculate the percentage of inhibition:

(5) $$I[\%]=\frac{\left(A0-As\right)}{A0}\times 100$$

where As is the sample’s absorbance and A0 is the absorbance of the negative control.

IC_50_ values were determined by interpolation from the concentration–response curve, using linear regression between inhibition percentages and extract concentrations. The IC_50_ was defined as the concentration required to inhibit 50% of DPPH radicals.

#### 4.4.2. Total Antioxidant Capacity (TAC)

The total antioxidant capacity of the extracts was assessed spectrophotometrically by the phosphomolybdenum method according to the procedure described by [22]. A total of 200 µL of each sample extract was mixed with 2 mL reagent solution (0.6 M H_2_SO_4_, 28 mM Sodium phosphate and 4 mM Ammonium molybdate). The mixtures were incubated at 95 °C for 90 to 120 min, and the absorbance was measured at 700 nm. A standard ascorbic acid calibration curve (0–200 µg/mL) was used for quantification, and the total antioxidant capacity (TAC) was expressed in milligrams of ascorbic acid equivalents per gram of dry matter (mg AAE/g DW).

### 4.5. HPLC-DAD Analysis

The chemoprofiling of the inflorescence extract of *F. communis,* obtained under the globally optimized extraction conditions predicted by the desirability function, was investigated using a reverse-phase high-performance liquid chromatography coupled with a diode array detector (HPLC-DAD). Before being analyzed by HPLC (Shimadzu, model [DGU-20A5R], Kyoto, Japan), the extracts (10 mg) were diluted in 1 mL of 80 percent methanol and filtered through 0.45 µm filters (Diode Array Detector) at a temperature of 40 °C, and Wakosil C18HG (5 mm, 4.6 × 150mm) was used to separate phenolic chemicals. Using a binary solvent combination made up of 50/50 methanol and acetonitrile and water acidified with 0.2 percent phosphoric acid (solvent A), the elution was performed in gradient mode (solvent B). After re-equilibrating for 12 min to the initial composition, a linear gradient was run from 96 percent (A) and 4 percent (B) to 50 percent (A) and 50 percent (B) for 40 min. It switched to 40 percent (A) and 60 percent for 5 min (B). It alternated between 0% (A) and 100% for 15 min (B). Each sample was injected at a volume of 20 µL at a flow rate of 1 mL/min for the mobile phase. The standard components, notably tannic acid, pyrogallol, gallic acid, catechol, hydroxytyrosol, caffeic acid, kaempferol, tyrosol, protocatechuic acid, syringic acid, coumaric acid, ferulic acid, vanillic acid, oleuropein, rutin, quercetin, and rosmarinic acid, were dissolved in HPLC-grade methanol. The phenolic components were identified through absorption spectra and retention time with the standard compounds used [23].

### 4.6. Experimental Design

The chosen experimental design was a central composite design with three factors. To ensure the reliability and estimate the pure experimental error, four replicates were performed at the central point of the domain, leading to a total of 17 experimental runs.

### 4.7. Mathematical Modeling

A second-order polynomial equation was postulated to model the relationship between the independent factors and the measured responses. The general form of the equation is:

Y = β_0_ + β_1_X_1_ + β_2_X_2_ + β_3_X_3_ + β_11_X_1_^2^ + β_22_X_2_^2^ + β_33_X_3_^2^ + β_12_X_1_X_2_ + β_13_X_1_X_3_ + β_23_X_2_X_3_ + ε (6)

In this model, Y represents the predicted response variable, corresponding to total phenolic content (TPC), total flavonoid content (TFC), DPPH IC_50_, or total antioxidant capacity (TAC). The term β_0_ denotes the intercept coefficient of the model, while β_1_, β_2_, and β_3_ represent the linear coefficients associated with the independent variables. The coefficients β_11_, β_22_, and β_33_ describe the quadratic effects, whereas β_12_, β_13_, and β_23_ account for the interaction effects between the studied factors. The variables X_1_, X_2_, and X_3_ correspond to the coded values of the independent factors, namely extraction time, ethanol concentration, and solvent-to-material ratio. Finally, ε represents the random error term of the model.

### 4.8. Statistical Analyses

The statistical significance of the developed models was evaluated using the F-test derived from analysis of variance (ANOVA). Student’s *t*-test was applied to assess the significance of the regression coefficients, while the goodness of fit of the models was evaluated using the coefficient of determination (R^2^). Statistical analyses were performed using JMP^®^ Pro software (version 17) and Design-Expert (version 12), which were employed for experimental design, data analysis, and optimization procedures. A confidence level of 95% was adopted, and *p*-values lower than 0.05 were considered statistically significant. The F-test was also used for comparing group means. Results are expressed as mean ± standard deviation (SD).

Measurements of total phenolic content (TPC), total flavonoid content (TFC), DPPH radical scavenging activity, total antioxidant capacity (TAC), and HPLC quantification were performed in triplicate as analytical replicates (*n* = 3). Results were expressed as mean ± standard deviation (SD).

## 5. Conclusions

The present study constitutes the first systematic investigation of the phenolic composition and antioxidant potential of *F. communis* inflorescence extracts using an optimized UAE-RSM approach, thereby filling a significant gap in the phytochemical characterization of this underexplored organ.

The optimization of extraction conditions by the central composite design method demonstrated that solvent polarity plays a determining role in the extraction yield of phenolic compounds. The hydroalcoholic mixture composed of 67% ethanol yielded the highest total polyphenol and flavonoid contents. This result underlines the importance of systematic parameter optimization over conventional approaches and provides a reproducible and transferable extraction protocol, directly applicable in laboratory and industrial settings.

The antioxidant activity results, obtained by two complementary methods the DPPH free radical scavenging test (IC_50_ = 0.17 mg/mL) and the total antioxidant capacity (23.6 mg AAE/g DW) confirmed the significant antioxidant potential of the studied extracts. The variations observed between the two methods reflect the chemical diversity of the antioxidant compounds present, with different phenolic fractions contributing distinctly to hydrogen atom transfer and electron transfer mechanisms.

The phenolic profile established by HPLC-DAD revealed a rich and diverse composition, with eleven identified compounds belonging to the hydroxycinnamic and hydroxybenzoic acid families. The predominance of kaempferol and pyrogallol provides a biochemical basis for the observed biological activities and supports the traditional medicinal uses attributed to this species. The simultaneous presence of less polar compounds such as ferulic acid, and vanillic acid further enriches the bioactive profile of the inflorescence and suggests a broad spectrum of potential biological activities worthy of further investigation.

Taken together, these results demonstrate that *F. communis* inflorescence extract exhibits a notable enrichment in phenolic compounds and a significant in vitro antioxidant potential. However, it should be emphasized that these findings are preliminary in nature, and that practical applications in the nutraceutical, pharmaceutical, and cosmetic sectors cannot be claimed at this stage. Further research is needed, including the complete identification and structural characterization of bioactive compounds, the validation of biological activities through in vitro and in vivo experimental models, and a thorough evaluation of the safety profile and potential toxicity of the extracts.

From a broader perspective, this work contributes to the valorization of a Mediterranean plant resource and opens several research avenues, including the in vitro and in vivo evaluation of specific biological activities, the identification of synergistic effects between the identified phenolic compounds, and the development of functional formulations based on standardized extracts. Future studies should also examine the seasonal and geographical variability of the phenolic composition, as well as the bioavailability and stability of the identified compounds under processing conditions.

## Figures and Tables

**Table 1:** Central composite design matrix with experimental results for TPC, TFC, DPPH-IC50, and TAC.

Run No.	Time (min)	ETOH Conc. (%)	Solvent-to-Solid Ratio	TPC (mg GAE/g DW)	TFC (mg QE/g DW)	DPPH-IC50 (mg/mL)	TAC (mg AAE/g DW)
1	14	40	10	11.59	8.20	6.39	14.84
2	14	40	30	11.67	10.69	6.43	14.43
3	14	80	10	18.97	10.86	0.65	14.29
4	14	80	30	20.70	12.86	1.99	14.54
5	30	40	10	17.63	14.03	2.64	18.35
6	30	40	30	15.63	12.06	3.51	16.14
7	30	80	10	19.63	15.11	1.46	15.76
8	30	80	30	20.10	15.29	2.62	13.19
9	14	60	20	23.03	13.89	2.26	18.40
10	30	60	20	28.47	16.07	1.38	20.99
11	22	40	20	22.77	13.75	3.21	23.21
12	22	80	20	32.52	15.63	0.58	19.59
13	22	60	10	26.10	16.63	0.72	21.36
14	22	60	30	28.07	17.41	1.64	21.24
15	22	60	20	34.93	17.36	1.40	21.99
16	22	60	20	30.07	17.36	1.65	23.86
17	22	60	20	32.47	18.03	1.64	24.04

TPC—Total Phenolic Content; TFC—Total Flavonoid Content; DPPH-IC50—DPPH radical scavenging capacity (IC_50_), half-maximal inhibitory concentration; TAC—Total Antioxidant Capacity.

**Table 2:** ANOVA results for the second-order models of TPC, TFC, DPPH-IC50, and TAC.

Response	Source of Variation	DF	Sum of Squares	Mean Square	F-Ratio	*p*-Value
TPC	Regression	9	796.24	88.47	25.79	0.0001
	Residual	7	24.02	3.43		
	Lack of fit	5	12.18	2.44	0.41	0.8169
	Pure Error	2	11.84	5.92		
	Total	16	820.26			
	R^2^	0.97				
	R^2^_adj_	0.93				
	R^2^_pred_	0.85				
TFC	Regression	9	121.61	13.51	44.08	<0.0001
	Residual	7	2.15	0.31		
	Lack of fit	5	1.85	0.37	2.47	0.1313
	Pure Error	2	0.30	0.15		
	Total	16	123.76			
	R^2^	0.98				
	R^2^_adj_	0.96				
	R^2^_pred_	0.77				
DPPH-IC50	Regression	9	47.12	5.24	36.11	<0.0001
	Residual	7	1.02	0.15		
	Lack of fit	5	0.97	0.19	9.73	0.0958
	Pure Error	2	0.04	0.02		
	Total	16	48.13			
	R^2^	0.98				
	R^2^_adj_	0.95				
	R^2^_pred_	0.85				
TAC	Regression	9	215.64	23.96	29.08	<0.0001
	Residual	7	5.77	0.82		
	Lack of fit	5	3.19	0.64	0.49	0.7731
	Pure Error	2	2.58	1.29		
	Total	16	221.41			
	R^2^	0.97				
	R^2^_adj_	0.94				
	R^2^_pred_	0.86				

**Table 3:** Estimated regression coefficients for the predictive models.

Term	Coeff.	TPC		TFC		DPPH-IC50		TAC	
		Estimate	*p*-Value	Estimate	*p*-Value	Estimate	*p*-Value	Estimate	*p*-Value
Intercept	β_0_	32.15	<0.0001 *	17.39	<0.0001 *	1.24	<0.0001 *	23.43	<0.0001 *
Time (X_1_)	β_1_	1.55	0.0330 *	1.61	<0.0001 *	−0.61	0.0014 *	0.79	0.0279 *
ETOH Conc. (X_2_)	β_2_	3.26	0.0008 *	1.10	0.0004 *	−1.49	<0.0001 *	−0.96	0.0124 *
S/M Ratio (X_3_)	β_3_	0.22	0.7126	0.35	0.0885	0.43	0.0087 *	−0.51	0.1216
X_1_ × X_2_	β_12_	−1.24	0.0994	−0.06	0.7524	1.01	0.0001 *	−0.64	0.0874
X_1_ × X_3_	β_13_	−0.42	0.5433	−0.79	0.0051 *	0.08	0.5645	−0.58	0.1116
X_2_ × X_3_	β_23_	0.51	0.4575	0.21	0.3249	0.20	0.1831	0.04	0.9103
X_1_^2^	β_11_	−6.15	0.0010 *	−2.26	0.0003 *	0.83	0.0093 *	−3.84	0.0002 *
X_2_^2^	β_22_	−4.26	0.0071 *	−2.55	0.0001 *	0.90	0.0062 *	−2.14	0.0063 *
X_3_^2^	β_33_	−4.82	0.0038 *	−0.22	0.5287	0.18	0.4536	−2.24	0.0050 *

* Statistically significant at the 95% confidence level (*p* < 0.05).

**Table 4:** Predicted and experimental values for the test point realized under optimal simultaneous parameters.

		TPC (mg GAE/g DW)		TFC (mg QE/g DW)		DPPH_IC50_ (µg/mL)		TAC (mg AAE/g DW)	
Parameters	Optimal Parameters Values	Predicted Value ^a^	Experimental Value ^b^	Predicted Value ^a^	Experimental Value ^b^	Predicted Value ^b^	Experimental Value ^a^	Predicted Value ^b^	Experimental Value ^a^
Time (min)	23	32.18 ± 1.87	31.66 ± 2.59	17.52 ± 0.56	18.26 ± 3.21	1.13 ± 0.38	0.95 ± 0.44	23.5 ± 0.92	21.75 ± 4.16
ETOH conc. (%)	60								
Solvent to material ratio	19								

^a^ The predicted value is given with the standard deviation of the response calculated from the model. ^b^ The observed value is the average of three replicates with standard error.

**Table 5:** HPLC analysis phenolic compounds in *F. communis* inflorescence extracts (mg/g extract).

Standards	RT	Inflorescence
**Pyrogallol**	3.41	1.05 ± 0.31
**Gallic acid**	5.41	0.53 ± 0.34
**Catechol**	6.24	0.22 ± 0.00
**kaempferol**	10.68	1.56 ± 0.13
**Protocatechuic acid**	12.54	0.28 ± 0.12
**Syringic acid**	13.25	0.16 ± 0.03
**Coumaric acid**	15.16	0.18 ± 0.17
**Ferulic Acid**	17.56	0.69 ± 0.12
**Vanillic acid**	18.56	0.51 ± 0.06
**Rutin**	22.47	0.93 ± 0.60
**Quercetin**	27.49	0.05 ± 0.06

Values are presented as mean ± standard deviation of three independent experiments (*n* = 3). RT: retention time.

## Data Availability

Data will be made available on request.
